# A comprehensive assessment of self-reported symptoms among patients harboring an unruptured intracranial aneurysm

**DOI:** 10.3389/fsurg.2023.1148274

**Published:** 2023-04-21

**Authors:** Ashia M. Hackett, Stefan W. Koester, Emmajane G. Rhodenhiser, Lea Scherschinski, Jarrod D. Rulney, Anant Naik, Elsa Nico, Adam T. Eberle, Joelle N. Hartke, Brandon M. Fox, Ethan A. Winkler, Joshua S. Catapano, Michael T. Lawton

**Affiliations:** Department of Neurosurgery, Barrow Neurological Institute, St. Joseph’s Hospital and Medical Center, Phoenix, AZ, United States

**Keywords:** cerebrovascular, microsurgical treatment, retrospective analysis, symptoms, unruptured intracranial aneurysms

## Abstract

**Background:**

Approximately 3.2%–6% of the general population harbor an unruptured intracranial aneurysm (UIA). Ruptured aneurysms represent a significant healthcare burden, and preventing rupture relies on early detection and treatment. Most patients with UIAs are asymptomatic, and many of the symptoms associated with UIAs are nonspecific, which makes diagnosis challenging. This study explored symptoms associated with UIAs, the rate of resolution of such symptoms after microsurgical treatment, and the likely pathophysiology.

**Methods:**

A retrospective review of patients with UIAs who underwent microsurgical treatment from January 1, 2014, to December 31, 2020, at a single quaternary center were identified. Analyses included the prevalence of nonspecific symptoms upon clinical presentation and postoperative follow-up; comparisons of symptomatology by aneurysmal location; and comparisons of patient demographics, aneurysmal characteristics, and poor neurologic outcome at postoperative follow-up stratified by symptomatic versus asymptomatic presentation.

**Results:**

The analysis included 454 patients; 350 (77%) were symptomatic. The most common presenting symptom among all 454 patients was headache (*n* = 211 [46%]), followed by vertigo (*n* = 94 [21%]), cognitive disturbance (*n* = 68[15%]), and visual disturbance (*n* = 64 [14%]). Among 328 patients assessed for postoperative symptoms, 258 (79%) experienced symptom resolution or improvement.

**Conclusion:**

This cohort demonstrates that the clinical presentation of patients with UIAs can be associated with vague and nonspecific symptoms. Early detection is crucial to prevent aneurysmal subarachnoid hemorrhage. It is imperative that physicians not rule out aneurysms in the setting of nonspecific neurologic symptoms.

## 1. Introduction

Approximately 3.2%–6% of the general population harbor an unruptured intracranial aneurysm (UIA). UIAs are increasingly found among women and are often detected between the fourth and sixth decades of life (1, 2). More than 91% of UIAs are asymptomatic and are only incidentally discovered when imaging is performed for unrelated reasons (3). Rupture of intracranial aneurysms represents a significant healthcare burden, with nearly 50% of cases resulting in death within 3 months of presentation. Of those patients who undergo life-saving treatment, 66% will experience varying degrees of permanent neurologic disability (4). Prevention of rupture, therefore, relies on early detection and treatment of aneurysms, although the clinical presentation is often nonspecific, and UIAs are frequently misdiagnosed on initial evaluation (5, 6). As such, recognition of symptoms related to UIAs and early detection are pertinent.

The International Study of Unruptured Intracranial Aneurysms (ISUIA) trial reported that the most common presenting symptoms are headache, ischemic cerebrovascular events, and cranial nerve deficits (7). Although headache is the most common reason for diagnostic imaging that leads to the detection of UIAs, it is unclear in most cases whether a headache is directly related to the aneurysm in the absence of subarachnoid hemorrhage (SAH) (2). Aneurysmal characteristics, such as size and location, can influence symptomatology and are likely responsible for compression of adjacent structures (1, 8). The pathophysiology associated with many of the vague symptoms reported in the presence of a UIA is not well understood. Additionally, many other nonspecific symptoms are associated with UIAs, which makes their diagnosis challenging. This study explores various symptoms associated with UIAs, the rate of resolution of such symptoms after microsurgical treatment, and the likely pathophysiology.

## 2. Methods

This retrospective observational cohort study was approved by the St. Joseph's Hospital and Medical Center Institutional Review Board (Phoenix, AZ) and complied with the Health Insurance Portability and Accountability Act. The need for patient consent was waived by the institutional review board due to the retrospective nature of the study.

All patients with a UIA who underwent microsurgical treatment between January 1, 2014, and December 31, 2020, at a single quaternary center were identified using a retrospective research database. Inclusion criteria were the availability of treatment data, adequate follow-up (≥6 weeks), and adequate symptom assessment. Patients who experienced recent (<6 months before surgery) SAH of an unrelated aneurysm were excluded from the analyses. Analyses included assessment of the prevalence of nonspecific symptoms upon clinical presentation and postoperative follow-up; comparisons of symptomatology by aneurysmal location; and a comparison of patient demographics, aneurysm characteristics, and poor neurologic outcome (defined as a modified Rankin Scale score greater than 2) at follow-up, with patients stratified by symptomatic versus asymptomatic presentation. Electronic medical records were analyzed for demographic information (age, sex, comorbidities), preoperative life expectancy, and Charlson Comorbidities Index. Aneurysm characteristics from computed tomography angiography were collected to assess each aneurysm's maximum diameter, neck diameter, perpendicular height (defined as the largest perpendicular distance from the neck of the aneurysm to the dome of the aneurysm), maximum height, aneurysm calcification, aneurysm type, location on critical perforating or branch vessels, intraluminal thrombosis, and size and aspect ratios. The size ratio was calculated as the maximum height divided by the mean vessel diameter of all branches associated with the aneurysm. The aspect ratio was calculated as the maximum perpendicular height divided by the neck diameter.

Statistical analyses included data aggregation, exploratory analysis, and multivariate analysis using R, version 4.0.1 (R Foundation for Statistical Computing). Demographic and clinical characteristics of patients were analyzed using a Wilcoxon rank sum test for interval variables, Pearson chi-square test for categorical variables, or Kruskal-Wallis rank sum test for nonparametric comparisons for multiple comparators. Fisher exact test was used for categorical variables to evaluate differences in asymptomatic and symptomatic patients and to compare symptoms based on aneurysm location. Significance was defined as *p* less than 0.05. Results are reported as mean (standard deviation [SD]) or as number (percentage) of patients.

## 3. Results

A total of 454 patients treated during the 7-year study period were included in the final analyses (Table 1). The mean (SD) age of all patients was 58 (12) years; 335 (74%) were women, and 119 (26%) were men. Most patients had a preoperative life expectancy of greater than 10 years (298 of 416 [72%]). The vascular categories involved included 152 (33%) middle cerebral artery, 116 (26%) anterior cerebral artery, 97 (21%) internal carotid artery (ICA), and 89 (20%) posterior circulation aneurysms. The mean (SD) aspect ratio was 1.72 (1.53) for 419 aneurysms, and 32 (7%) of 434 aneurysms had calcification. Overall, 90% (*n* = 408) aneurysms were saccular and the remaining 10% (*n* = 46) were nonsaccular. Additionally, 126 (29%) of 431 UIAs were found on critical perforating vessels or vessel branches.

**Table 1:** Patient demographics and aneurysm characteristics among 454 patients with unruptured intracranial aneurysms.

Characteristic	Value, *N* = 454^a^
Sex	
Female	335 (74)
Male	119 (26)
Age, mean (SD), years	58 (12)
Symptomatic	350 (77)
Prior SAH	50 (11)
Preoperative life expectancy (*n* = 416)	
>10 years	298/416 (72)
5–10 years	114/416 (27)
<5 years	4/416 (1)
Comorbidities	
Anxiety	30 (7)
Depression	42 (9)
Diabetes mellitus	71 (16)
Hypercholesterolemia	140 (31)
Hyperlipidemia	41 (9)
Hypertension	281 (62)
Charlson Comorbidity Index	
0	97 (21)
1	85 (19)
2	115 (25)
3	94 (21)
4	42 (9)
5	16 (4)
6	4 (1)
7	0 (0)
8	1 (0.2)
Vasculature location	
ACA	116 (26)
ICA	97 (21)
MCA	152 (33)
Posterior circulation	89 (20)
Aneurysm measurements, mean (SD)	
Maximum diameter, mm (*n* = 446)	6.29 (4.63)
Maximum perpendicular height, mm	5.12 (3.38)
Maximum height, mm	5.48 (3.55)
Maximum neck diameter, mm	3.66 (1.37)
Aspect ratio (*n* = 419)	1.72 (1.53)
Size ratio	2.52 (1.39)
Aneurysm characteristics	
Calcification (*n* = 434)	32 (7)
Complex saccular	60 (13)
Critical or perforating branch vessels (*n* = 431)	126 (29)
Intraluminal thrombosis on imaging (*n* = 433)	18 (4)
Nonsaccular	46 (10)
Saccular	408 (90)

ACA, anterior cerebral artery; ICA, internal carotid artery; MCA, middle cerebral artery; SAH, subarachnoid hemorrhage; SD, standard deviation.

^a^ Data are presented as number (%) of 454 observations unless otherwise noted.

Overall, 350 (77%) of the 454 patients were symptomatic; some patients had more than 1 symptom. The most common presenting symptom was headache, occurring in 211 (46%) patients, followed by vertigo (94 [21%]), cognitive disturbance (68 [15%]), and visual disturbance (64 [14%]) (Table 2). Of 328 patients for whom symptom resolution or improvement was assessed, 258 (79%) experienced full symptom resolution or improvement (Table 3). Of 204 patients assessed for headache resolution, 58 (28%) experienced residual headache at follow-up. Of these 58 patients, 32 (55%) reported an ability to control headache with over-the-counter pain medication. Of 23 patients assessed for cranial nerve recovery, 4 (17%) experienced residual cranial nerve deficits.

**Table 2:** Presenting symptoms among 454 patients with unruptured intracranial aneurysms.

Characteristic	No. (%) of patients, *N* = 454
Cognitive disturbance	68 (15)
Cranial nerve deficit	25 (6)
Gait imbalance	58 (13)
Headache	211 (46)
Hearing disturbance	9 (2)
Ischemic event	17 (4)
Limb weakness	29 (6)
Nausea and/or vomiting	22 (5)
Paresthesia	35 (8)
Seizure	10 (2)
Syncope/near syncope	24 (5)
Tinnitus	3 (1)
Vertigo	94 (21)
Visual disturbance	64 (14)
Other	15 (3)

**Table 3:** Symptoms reported at postoperative follow-up among 454 patients with microsurgical treatment of unruptured intracranial aneurysms.

Characteristic	No. of patients with symptom/no. of observations (%)
Symptoms completely resolved and/or improved	258/328 (79)
Residual headache	58/204 (28)
Headache controlled by over-the-counter medication	32/58 (55)
Residual vertigo	17/94 (18)
Residual visual disturbance	10/64 (16)
Residual cognitive disturbance	10/62 (16)
Residual cranial nerve deficit	4/23 (17)

Overall, 104 (23%) patients were asymptomatic and 350 (77%) were symptomatic at presentation (Table 4). The proportion of patients with symptomatic presentation was greater among women (266/335 [79%]) than among men (84/119 [71%]). Compared with asymptomatic presentation, symptomatic presentation was associated with a greater prevalence of hypertension (65% [228/350] vs. 51% [53/104]; *p* = 0.009), clinically diagnosed anxiety (8% [29/350] vs. 1% [1/104]; *p* = 0.008], and diabetes mellitus (18% [62/350] vs. 9% [9/104]; *p* = 0.03). A significantly greater proportion of patients with asymptomatic presentations than patients with symptomatic presentations had an SAH >6 months before microsurgical treatment of a UIA (21% [22/104] vs. 8% [28/350]; *p* ≤ 0.001). The mean (SD) maximum aneurysm diameter was significantly greater in the symptomatic cohort than in the asymptomatic cohort (6.54 [4.98] mm vs. 5.46 [3.06] mm, *p* = 0.004). The proportion of patients with poor neurologic outcome (modified Rankin Scale score >2) at postoperative follow-up was not significantly different between the symptomatic and asymptomatic cohorts (14% [50/350] vs. 10% [10/104]; *p* = 0.15).

**Table 4:** Comparison of demographic and clinical characteristics of 104 asymptomatic and 350 symptomatic patients with unruptured intracranial aneurysms.

Characteristic^a^	Asymptomatic, *N* = 104	Symptomatic, *N* = 350	*p* value*
Sex			0.049
Female	69 (66)	266 (76)	
Male	35 (34)	84 (24)	
Age, mean (SD), years	59 (13)	58 (12)	0.09
Prior SAH	22 (21)	28 (8)	<0.001
Preoperative life expectancy (*n* = 416)			0.51
>10 years	73/96 (76)	225/320 (70)	
5–10 years	22/96 (23)	92/320 (29)	
<5 years	1/96 (1)	3/320 (1)	
Comorbidities			
Anxiety	1 (1)	29 (8)	0.008
Depression	6 (6)	36 (10)	0.16
Diabetes mellitus	9 (9)	62 (18)	0.03
Hypercholesterolemia	33 (32)	107 (31)	0.82
Hyperlipidemia	9 (9)	32 (9)	0.88
Hypertension	53 (51)	228 (65)	0.009
Vasculature location			0.03
ICA	12 (12)	85 (24)	
ACA	34 (33)	82 (23)	
MCA	37 (36)	115 (33)	
Posterior circulation	21 (20)	68 (19)	
Maximum diameter, mean (SD), mm (*n* = 446)	5.46 (3.06)	6.54 (4.98)	0.004
Aspect ratio, mean (SD) (*n* = 419)	1.56 (0.61)	1.76 (1.71)	0.11
Aneurysm calcification (*n* = 434)	7 (7)	25 (8)	0.85
Nonsaccular	5 (5)	41 (12)	0.04
Saccular	99 (95)	309 (88)	0.04
Complex saccular	16 (15)	44 (13)	0.46
Critical or perforating branch vessels (*n* = 431)	24 (24)	102 (31)	0.21
Intraluminal thrombosis on imaging (*n* = 433)	4 (4)	14 (4)	>0.99
Death	3 (3)	12 (3)	>0.99
Discharge mRS score (*n* = 452)			0.83
1	37/103 (36)	106/349 (30)	
2	23/103 (22)	92/349 (26)	
3	37/103 (36)	131/349 (38)	
4	6/103 (6)	17/349 (5)	
5	0/103 (0)	2/349 (1)	
6	0/103 (0)	1/349 (0.3)	
Follow-up mRS score			0.048
0	27/104 (26)	63/350 (18)	
1	51/104 (49)	181/350 (52)	
2	16/104 (15)	56/350 (16)	
3	1/104 (1)	27/350 (8)	
4	6/104 (6)	12/350 (3)	
5	0 (0)	0 (0)	
6	3/104 (3)	11/350 (3)	
Follow-up mRS score >2	10 (10)	50 (14)	0.15

ACA, anterior cerebral artery; ICA, internal carotid artery; MCA, middle cerebral artery; mRS, modified Rankin Scale; SAH, subarachnoid hemorrhage; SD, standard deviation.

^a^ Data are presented as number (%) of patients unless otherwise noted.

* Pearson chi-square test, Wilcoxon rank sum test, or Fisher exact test used to compare groups.

Table 5 reports symptoms by vascular territory. The proportion of patients with intracranial internal carotid artery aneurysms who were symptomatic (85 of 97 [88%]) was significantly greater than the proportion of patients with aneurysms in other locations who were symptomatic (*p* = 0.03). Gait imbalance (19 of 97 [20%]; *p* = 0.04) and limb weakness (13 of 97 [13%]; *p* = 0.01) were both significantly more prevalent in patients harboring an intracranial ICA aneurysm, compared with patients with aneurysms in other vascular locations. The prevalence of cranial nerve deficit was comparable in patients with ICA aneurysms (11 of 97 [11%]) and patients with posterior circulation aneurysms (11 of 97 [11%]); the prevalence of cranial nerve deficit among these patients was significantly greater than the prevalence among patients with aneurysms at other locations (*p* ≤ 0.001). The highest burden of cognitive changes was found among patients with aneurysms in the middle cerebral artery territory (29 of 152 [19%]; *p* = 0.04).

**Table 5:** Symptoms among 454 patients with unruptured intracranial aneurysms, by aneurysm location.

Characteristic	No. (%) of patients				*p* value*
	ICA, *N* = 97	ACA, *N* = 116	MCA, *N* = 152	Posterior circulation, *N* = 89	
Cognitive disturbance	17 (18)	17 (15)	29 (19)	5 (6)	0.04
Cranial nerve deficit	11 (11)	2 (2)	2 (1)	10 (11)	<0.001
Gait imbalance	19 (20)	13 (11)	21 (14)	5 (6)	0.04
Headache	54 (56)	47 (41)	74 (49)	36 (40)	0.09
Hearing disturbance	4 (4)	1 (1)	3 (2)	1 (1)	0.44
Ischemic event	7 (7)	3 (3)	6 (4)	1 (1)	0.17
Limb weakness	13 (13)	3 (3)	9 (6)	4 (5)	0.01
Nausea and/or vomiting	4 (4)	6 (5)	6 (4)	6 (7)	0.76
Paresthesia	9 (9)	9 (8)	10 (7)	7 (8)	0.89
Tinnitus	1 (1)	0 (0)	2 (1)	0 (0)	0.62
Seizure	2 (2)	3 (3)	5 (3)	0 (0)	0.40
Symptomatic	85 (88)	82 (71)	115 (76)	68 (76)	0.03
Syncope or near syncope	6 (6)	6 (5)	8 (5)	4 (5)	0.97
Vertigo	20 (21)	21 (18)	32 (21)	21 (24)	0.82
Visual disturbance	20 (21)	17 (15)	13 (9)	14 (16)	0.06
Other	5 (5)	2 (2)	4 (3)	4 (5)	0.44

ACA, anterior cerebral artery; ICA, internal carotid artery; MCA, middle cerebral artery.

* Pearson chi-square test, Kruskal-Wallis rank sum test, or Fisher exact test used to compare groups.

## 4. Discussion

In our retrospective cohort, 77% (350/454) of patients presented with symptoms; often, aneurysms were discovered during evaluation for unrelated issues. It is unclear whether symptoms were a direct result of the aneurysms, yet 79% of the patients who had follow-up of 6 weeks or greater reported either improvement or complete resolution of presenting symptoms after microsurgical treatment. Although the general population of patients with UIAs tends to be asymptomatic, our institution often receives referrals of patients with complex or atypical presentations, which may account for the high percentage of patients who presented with symptoms. Patients with prior SAH (>6 months prior) were significantly more likely than other patients to have an asymptomatic presentation associated with their unruptured aneurysm diagnosis, likely due to recommended follow-up imaging after experiencing an aneurysmal SAH (aSAH). Interestingly, the presence of a clinically diagnosed anxiety disorder, prior to aneurysm diagnosis, was associated with a symptomatic UIA. Although the relationship between anxiety and aneurysms after SAH and treatment has been explored, the relationship between anxiety and symptomatic UIAs has not been discussed in literature (9). A possible explanation for this association is that patients with nonspecific symptoms who experience anxiety may be more persistent with follow-up and request further investigation, leading to higher rates of incidental UIA findings.

Headaches are the most frequently reported symptom of UIAs in the literature (10, 11) and were reported in 46% (211/454) of patients in our cohort. In these patients, the presence of an aneurysm is often missed due to nonspecificity and the varied characterization of headaches associated with UIA. Headaches can present as chronic with variable characterization in two-thirds of cases, but they may also present acutely as a sudden onset headache in one-third of cases (11). Although acute “thunderclap” headaches are typical of aSAH, less severe and more chronic headaches may be associated with UIAs. For example, a “sentinel headache” is well-described in the literature and is thought to be associated with local thrombosis (12). Chronic headaches may have variable presentations but most frequently resemble migraines (13). In 2013, Lebedeva et al. (14) conducted a prospective case-control study of 199 patients and found that migraine-like headaches without aura were significantly associated with saccular aneurysms up to 1 year before rupture (odds ratio [OR], 6.7; 95% confidence interval [CI], 3.8–11.9; *p* ≤ 0.001). Another examination of 172 patients found that a history of migraines was significantly associated with UIAs (OR, 1.9; 95% CI, 1.1–3.5) but no other headache types (15). Although the pathophysiology of headaches among patients with UIAs is a topic of ongoing research, headaches most likely arise from local inflammatory processes involving the meninges or cranial nerves, pulsation of the aneurysm itself, or local thrombosis (11). Intriguingly, headaches have been found to resolve in the majority of patients after treatment (11, 16, 17). Although headache was the most common residual symptom at follow-up in our analysis, 55% (32/58) of those patients reported an ability to control the pain with over-the-counter medications.

Only patients with a clear diagnosis of a cranial nerve palsy were categorized into the cranial nerve deficit cohort in this analysis. However, patients reporting hearing or visual disturbances could be experiencing a cranial nerve deficit as well. Specifically, aneurysms of the anterior inferior cerebellar artery have been implicated in patients presenting with symptoms of hearing loss and tinnitus, likely due to the close approximation of the artery to the internal auditory canal as well as cranial nerves VII and VIII (18, 19). In our study, 3 patients presented with an aneurysm of the anterior inferior cerebellar artery, and 1 of these patients had a cranial nerve deficit.

A range of visual deficits were reported in our patient cohort (14% [64/454]). Aneurysm-related visual changes commonly reported in the literature may include diplopia, ptosis, pupillary dilation, and lateral deviation of the affected eye, likely secondary to oculomotor palsy. Decreased visual acuity as a result of optic pathway compression has also been reported. In fact, 2 of the most common causes of non–pupil-sparing oculomotor nerve palsy are posterior communicating artery aneurysms and distal basilar artery aneurysms (20). Less commonly, aneurysms of the cavernous portion of the ICA have been reported to cause third cranial nerve palsy (21). Although the pathophysiology behind oculomotor nerve palsy is thought to be due to pulsatility and compressive mass effect of large aneurysms, there are reports of small aneurysms causing third cranial nerve palsy, refuting the idea that these deficits are strictly related to aneurysm size (21–23). Any aneurysm of the circle of Willis can cause anterior optic pathway compression and result in visual deficits; however, the most common locations include the ICA, specifically the paraclinoid region. In a study conducted by Park et al. (8), the most common aneurysms causing visual deficits arose from the ophthalmic segment of the ICA (31 of 33 cases). The close approximation of the ICA to the optic nerve and optic chiasm may yield compression from UIAs and is likely the cause of visual disturbances. Direct compression is thought to be the most common etiology; however, other causes include diminished blood supply due to compromise of the ophthalmic artery or small arterial branches in the parasellar and suprasellar regions (24–26).

In the ISUIA trial, the second most common symptom reported among patients presenting with UIAs was an ischemic cerebrovascular event (2). In our cohort of 454 patients, 17 (4%) presented with signs and symptoms of either a transient ischemic attack or cerebrovascular infarction, and an aneurysm was discovered upon further investigation. Both hemodynamic and biological factors of aneurysms contribute to the prothrombotic environment within the aneurysmal sac, which may result in subsequent thrombosis of the parent vessel or dislodged emboli from the thrombus (27). Compression of nearby vasculature can also result in ischemic events. In a retrospective study of 3,202 patients, reported by Calviere et al. (28), 15 patients (0.47%) were found to have stroke or transient ischemic attack solely caused by UIAs, among whom 10 had evidence of aneurysmal thrombosis. Aneurysmal thrombosis was significantly associated with ischemic stroke (*p* = 0.02) in that study. Additionally, cases of large vessel occlusion caused by a thrombus formed in the aneurysmal sac have been documented in the literature (29–31). Although aneurysms can be an exclusive cause of ischemia, a UIA discovered in the presence of an ischemic event is usually thought to be an incidental finding due to the overlapping risk factors of both strokes and aneurysms, which include hypertension, diabetes, hyperlipidemia, and smoking (28, 32). As such, UIAs are more commonly found in patients who have experienced an ischemic stroke than in the general population. In our cohort, among patients with hypertension and patients with diabetes, the proportion who were symptomatic at presentation was greater than the proportion who were asymptomatic at presentation.

Vertigo was the second most common presenting symptom (21% [94/454]) in our UIA cohort. In 1 case reported by Oh et al. (33), a patient who was later found to have a large left vertebral artery aneurysm had a clinical presentation consisting of positional vertigo and vomiting, which resolved after aneurysm resection. The likely cause of this patient's clinical presentation was presumed to be secondary to mass effect on the inferior cerebellum around the fourth ventricle and compression of the area postrema. In another case, a patient presented with headache, nausea, vomiting, and vertigo; an unruptured aneurysm, partially eroding the floor of the sella and causing hydrocephalus, was identified (34). Cerebral aneurysms of the ICA and anterior communicating artery can mimic sellar lesions; in the reported case, cerebral angiography identified an aneurysm of the right carotid artery at the intracavernous tract.

The functional disability of people with seizures often prompts further workup, yet it is unclear whether seizures are a direct result of aneurysms or incidental findings. In our analysis, only 2% (10/454) of the patients had an aneurysm that was discovered upon workup for seizures. However, a series of articles have reported seizure as a primary presenting symptom of UIA (12, 35–38). Of 662 surgically managed unruptured intracranial aneurysms, Patil et al. (38) reported a total of 3 patients with unruptured anterior communicating artery aneurysms who presented with seizures as the only symptom. In a separate study, Hanggi et al. (39) assessed 347 UIA patients, and 9 presented with seizures, all of which resolved after aneurysm treatment. In the same study, a comprehensive review of seizures secondary to aneurysms suggested direct or intermittent cortical compression can cause cortical gliosis and resultant epileptogenesis. The authors concluded that surgical resection of the surrounding gliosis leads to a seizure-free postoperative course (39).

Additional and extremely vague symptoms displayed in our analysis of 454 patients included syncope and near syncope (24 [5%]), limb weakness (29 [6%]), paresthesia (35 [8%]), gait imbalance (58 [13%]), and cognitive-related impairments (68 [15%]). Many of these symptoms are nonspecific, and reports of them in the literature are limited to case studies. Syncope was identified in 1 other case of a woman with transient syncopal episodes prompting presentation to the emergency department, at which time an unruptured fusiform mid-basilar artery aneurysm was incidentally found (40). A case report published in 2021 details a patient with a giant thrombosed middle cerebral artery aneurysm, with gait disturbance as the sole presenting symptom (41). A case series published in 1980 described 3 patients with episodic weakness and numbness of the arms and legs and 1 patient with confusion spells leading to incidental findings of UIAs; 3 of the 4 patients reported symptom resolution after treatment (42). It is unclear whether these extremely vague symptoms are a direct result of the aneurysms, and a direct correlation remains difficult to assess.

Limitations of this study include its retrospective design and an inability to account for all confounding variables. Additionally, the external validity of this study was limited because it was conducted at a single institution that receives high volumes of patient referrals with atypical and complex clinical presentations. Furthermore, it is difficult to ascertain whether clinical symptoms upon presentation are related to the aneurysm or whether the aneurysm is an incidental finding.

## 5. Conclusion

The clinical presentation of patients with UIAs can consist of vague and nonspecific symptoms. In the setting of nonspecific neurologic symptoms, such as headache, cranial nerve deficits, ischemic events, and even seizures, UIAs should be considered as a potential etiology. In many cases, it is unclear whether aneurysms cause these symptoms or are simply an incidental finding. However, a substantial proportion of patients with UIAs experience resolution of nonspecific symptoms after aneurysm treatment. Aneurysms may therefore be the origin of these symptoms through varied pathogenesis. Because early detection is crucial to prevent aSAH, it is imperative that physicians not rule out aneurysms in the setting of nonspecific neurologic symptoms.

## Data Availability

The raw data supporting the conclusions of this article will be made available by the authors, without undue reservation.
